# Cabazitaxel versus docetaxel for treatment of metastatic castrate refractory prostate cancer

**DOI:** 10.1002/bco2.177

**Published:** 2022-06-18

**Authors:** Nicholas D. James, Ayesha Ali, Ann Pope, Amisha Desai, Daniel Ford, Robert Stevenson, Anjali Zarkar, Sarah Pirrie, the CANTATA investigators

**Affiliations:** ^1^ Institute of Cancer Research London UK; ^2^ Cancer Research UK Clinical Trials Unit (CRCTU) University of Birmingham Birmingham UK; ^3^ University Hospital Birmingham Cancer Centre, University Hospital Birmingham Birmingham UK

**Keywords:** cabazitaxel, clinical trial, docetaxel, metastatic castrate refractory prostate cancer, phase II trial

## Abstract

**Objectives:**

To assess cabazitaxel versus docetaxel re‐challenge for the treatment of metastatic castrate refractory prostate cancer (CRPC) patients previously treated with docetaxel at inception of primary hormone therapy.

**Patients and Methods:**

The CANTATA trial was a prospective, two‐arm, open‐label, phase II study conducted in eight UK centres. Patients over the age of 18, with histologically proven, metastatic prostate cancer who had been previously treated with up to 6 cycles of docetaxel as part of the STAMPEDE trial (or treated with the same drug outside of the trial at primary diagnosis) and had a performance status (PS) of 0–2, were eligible. Patients who progressed during primary treatment with docetaxel or had received prior systemic chemotherapy were excluded. Cabazitaxel (25 mg/m^2^) or docetaxel (75 mg/m^2^) was administered via intravenous infusion every 3 weeks with oral prednisolone (10 mg) for up to 10 cycles, until disease progression, death or unacceptable toxicity. The primary outcome was clinical progression‐free survival (PFS) as defined by either date of pain progression, date of a cancer‐related skeletal‐related event, or date of death from any cause. Analyses were by intention to treat. EudraCT number: 2012‐003835‐40

**Results:**

Between 7 March 2013 and 4 January 2016, 15 patients with a median age of 70 years (range 54–76) were recruited; seven received cabazitaxel, eight docetaxel. The study was halted due to slow accrual. The median clinical PFS time in the cabazitaxel group was 6.2 months compared with 8.4 for the docetaxel group (95% confidence intervals were not reached due to the small number of patients). A total of 13 serious adverse events were reported.

**Conclusion:**

Due to the low number of patients recruited, meaningful comparisons could not be made. However, toxicity was in line with known outcomes for these agents, demonstrating it is feasible and safe to deliver chemotherapy to men relapsing with CRPC after upfront chemotherapy.

## 1 INTRODUCTION

Prostate cancer is a major worldwide health problem and represents 12% of all diagnosed cancers in the UK. Between 2014 and 2016, 10 187 men were diagnosed with prostate cancer, of whom 5407 died from the disease between 2015 and 2017 in the United Kingdom.^1^ The first‐line treatment for locally advanced or metastatic prostate cancer is androgen deprivation, either surgically via bilateral orchidectomy or medically with LHRH analogues or an anti‐androgen.^2^ Hormone therapy is not curative, and all patients will become refractory to standard hormone therapies, with a median time to progression of 18–24 months on first‐line treatment, at which point the prognosis becomes poor; historically median survival was 7–15 months.^3^ Over the last two decades, a range of treatments have been licensed for what used to be called hormone‐refractory prostate cancer (HRPC) but which is increasingly referred to as castrate refractory prostate cancer (CRPC). These therapies include abiraterone,^4^, ^5^ enzalutamide,^6^, ^7^ radium‐223,^8^ docetaxel^9^, ^10^ and cabazitaxel,^11^ with other therapies in advanced stages of development.

The STAMPEDE,^12^ CHAARTED,^13^ GETUG‐12^14^ and GETUG‐15^15^ trials investigated the upfront use of docetaxel and showed a clear benefit on a range of outcomes in metastatic disease, including overall survival.^16^ The use of upfront docetaxel, therefore, raised the question of which taxane chemotherapy to use in men after relapse with metastatic (m)CRPC.

Surveys of the STAMPEDE investigators suggested that trial patients relapsing in the docetaxel arm would be offered re‐challenge with docetaxel at relapse, unless there was clear evidence of docetaxel resistance (i.e., disease progression whilst on chemotherapy). If the position of docetaxel in the treatment pathway is moved to the upfront setting in high‐risk disease, the question arises as to which chemotherapy drug should be used in the metastatic castrate resistant setting in patients who received primary docetaxel.

The TROPIC trial compared cabazitaxel with mitoxantrone in patients previously treated with docetaxel and has shown a statistically significant survival advantage with cabazitaxel.^11^ On the basis of this trial, cabazitaxel is now licensed for use after failure of docetaxel in mCRPC. Cabazitaxel was, however, associated with a range of typical chemotherapy side effects. Of these, myelosuppression was prominent. The mean number of prior docetaxel chemotherapy cycles in TROPIC was around nine, and the median gap between first‐ and second‐line treatments was approximately three months. In contrast, the median time to relapse in the STAMPEDE trial control arm is around two years, and the maximum chemotherapy exposure is six cycles. Therefore, although still second‐line chemotherapy, the patient exposure to prior chemotherapy, the recovery interval and hence the likely risks of toxicity are much more favourable.

In light of the positive results from the TROPIC trial, there was a unique opportunity to investigate the role of cabazitaxel in relapsing mCRPC patients treated with primary docetaxel. Therefore, the aims of the CANTATA trial were to establish whether cabazitaxel can benefit clinical PFS compared with docetaxel re‐challenge as second‐line chemotherapy treatment in patients with mCRPC, whilst being weary of cabazitaxel's significant haematological toxicities. The data gained from the proposed randomised phase II trial could have provided an ideal platform for a larger phase III trial with the HRPC docetaxel studies supporting a change to the use of docetaxel within high‐risk and metastatic prostate cancer at diagnosis.

## 2 PATIENTS AND METHODS

### 2.1 Study design

The CANTATA trial was a two‐arm, randomised, non‐blinded phase II clinical trial recruiting patients from eight hospitals in the United Kingdom. Ethical approval for the trial protocol (ultimately Version 3.0 dated 27 June 2014) was obtained from West Midlands Research Ethics Committee and local institutional review boards and ethical committees in accordance with national and international guidelines.

### 2.2 Patients

Patients aged 18 or over, with histologically proven prostate adenocarcinoma that was castrate refractory, who had been previously treated with up to s6 cycles of docetaxel as part of the STAMPEDE trial (or treated with the same drug outside of the trial at primary diagnosis), with confirmed biochemical, radiological or clinical progression and metastatic disease, with a PS of 0–2, adequate bone marrow, hepatic and renal function, and were available for long‐term follow‐up (minimum of 2 years), were eligible for this trial. Patients with prior systemic therapy with chemotherapy other than docetaxel, who had progressive disease whilst on primary docetaxel, with metastatic brain or leptomeningeal disease, active (Grade ≥2) peripheral neuropathy or were receiving systemic antibiotic or anti‐fungal medication(s), were excluded. Patients with reproductive potential were required to use effective methods of contraception. All patients gave written informed consent for the trial and the optional quality of life (QoL) substudy.

### 2.3 Randomisation and masking

Eligible patients were randomised (1:1) to receive cabazitaxel or docetaxel. Treatment allocation was by a randomised scheme loaded into the Interactive Web Recognition System (IWRS) database at the Cancer Research UK Clinical Trials Unit (CRCTU) at the University of Birmingham. Randomisation was stratified by prior exposure to albiratetone, enzalutamide (MDV3100) or other new generation hormone therapies (e.g., TAK700) and balanced within treatment centres.

### 2.4 Procedures

Cabazitaxel was administered at a dose of 25 mg/m^2^ (in either a 0.9% sodium chloride or 5% dextrose solution) as a 1 hour intravenous (i.v.) infusion every 3 weeks in combination with daily oral prednisolone (10 mg). Cabazitaxel was administered in an outpatient setting. Prior to cabazitaxel administration, a 30 minute i.v. antihistamine (chlorpheniramine 5 mg or equivalent), dexamethasone 8 mg or equivalent, and H2 agonist (ranitidine 500 mg or equivalent) was administered. Antiemetic prophylaxis was also recommended with variations in all pre‐treatments permitted in discussion with the CANTATA Trial Office. Additional treatment of granulocyte colony‐stimulating factor was recommended for those patients with severe neutropenia or neutropaenic sepsis.

Docetaxel was administered at a dose of 75 mg/m^2^ (in either a 0.9% sodium chloride or 5% dextrose solution) as a 1 hour i.v. infusion every 3 weeks in combination with daily oral prednisolone (10 mg). Pre‐medication with oral dexamethasone (8 mg) was recommended, as well as antiemetic regimens 30 minutes before docetaxel of ondansetron (8 mg i.v.) with dexamethasone (8 mg i.v.) followed by ondansetron (8 mg) for 3 days with domperidone (20 mg).

New cycles of cabazitaxel or docetaxel did not begin until the neutrophil and platelet counts had reached ≥1.5 × 10^9^ L or 100 × 10^9^ L, respectively, with non‐haematological toxicities (except alopecia) restored to baseline levels. Patients were treated until disease progression, death, unacceptable toxicity or up to a maximum of 10 cycles.

Pre‐treatment evaluation included the following: CT or MRI pelvis and abdomen scan, bone scan and a chest X‐ray if area not included in CT, as well as blood biochemistry, prostate‐specific antigen (PSA) assessment, and liver and kidney function tests. CT scans were performed when clinically indicated with additional blood biochemistry, PSA, and liver and kidney tests performed within 7 days of first treatment, at the start and end of each cycle treatment, and at each follow‐up visit. Adverse events according to NCI‐CTCAE v4.03^17^ were recorded at the start and end of each cycle and at each follow‐up visit. Follow‐up data were collected at standard post‐treatment clinic visits at approximately 3‐monthly intervals for up to 2 years. QoL questionnaires were administered by research nurses prior to randomisation, on day 30 of each treatment cycle and at every follow‐up visit (typically 3‐monthly). They were completed independently by patients.

### 2.5 Outcomes

The protocol‐defined primary outcome was to determine the activity of cabazitaxel compared with docetaxel re‐challenge as second‐line chemotherapy treatment by measuring clinical PFS. Clinical progression was defined as the earliest date of pain progression (the date a patient was seen in clinic and pain progression identified), date of occurrence of a cancer‐related skeletal‐related event (SRE), or date of death from any cause.

The secondary objectives were to assess the toxicity profile and rate of toxicities associated with the study treatment. SRE‐free survival was defined as the time in whole days from the date of randomisation to the date of the first occurrence of an SRE. An SRE was defined as any one of the following; symptomatic pathological bone fracture; spinal cord or nerve root compression likely to be related to cancer or treatment; cancer related surgery to bone; radiation therapy to bone (including use of radioisotopes); change of anti‐neoplastic therapy to treat bone pain due to prostate cancer; or hypercalcaemia. Patients who did not experience a SRE were censored at death or the date last known to be alive. Pain PFS was defined as the time in whole days from the date of randomisation to the date of clinician‐determined pain progression. Patients not experiencing pain progression were censored at the date of death or their last known to be alive date. PSA PFS applied only to patients with baseline PSA (20 ng/ml). A response required a PSA decline of 50% confirmed by a second PSA value at least 3 weeks later. The duration of PSA response was measured from the first to the last assessment at which the above criteria were satisfied. ‘Best’ PSA response during treatment, and prior to progression, contributed to the primary outcome. PSA PFS was defined as the time from the start of initial treatment to the progression of PSA. Patients who did not experience PSA progression were censored at their date of death, or at the date, they were last known to be alive. QoL was assessed using two validated instruments: the European Organisation for Research and Treatment of Cancer (EORTC) QLQ‐C30 for cancer^18^ and the prostate‐specific EORTC QLQ‐PR25.^19^

### 2.6 Statistical analysis

The sample size calculation for the CANTATA trial was based on a Jung's Single Stage design^20^ and the primary outcome measure of clinical PFS at 6 months. At study conception, evidence suggested that patients receiving docetaxel had a response rate of 20%. The design is based on ensuring the type I and II error rates are ≤0.10 and ≤0.15, respectively, and assumes a response rate on the control arm (docetaxel) of 20% with a 15% expected absolute improvement in the treatment arm (cabazitaxel). This design requires 65 patients to be randomised to each treatment arm, with target recruitment of 69 patients per arm (138 total) to allow for a 5% drop‐out rate to provide evidence that cabazitaxel treatment warrants further investigator in a phase III setting.

Kaplan–Meier curves were created and used to estimate clinical PFS percentages at median follow‐up and 6 months, along with 95% confidence intervals (CIs). Analysis of clinical PFS was carried out using an adjusted approach; the first analysis an adjusted log‐rank test, comparing cabazitaxel and docetaxel, stratified by prior hormone therapy and presence of pain at randomisation. Conclusions are based on a two‐sided 5% significance level. No adjustments for multiple testing were made. The second analysis used an adjusted Cox regression model, including both the treatment comparison and stratification factors. The use of both the log‐rank and Cox regression models was pre‐specified in the statistical analysis plan. No model selection techniques were employed. A subgroup analysis was also specified, to assess treatment effects separately in patients with and without exposure to new generation hormone therapies and in patients with and without pain but could not be carried out due to the lower number of events. All analyses were conducted on an intention‐to‐treat basis, with analyses performed in R.

An independent Data Monitoring Committee reviewed interim data annually to ensure patient safety, recruitment rate and data quality. There were no formal stopping rules. The trial was registered on the EU Clinical Trials Register with EudraCT number 2012‐003835‐40.

### 2.7 Role of the funding source

The trial was sponsored by University of Birmingham and run by the CRCTU located there. Funding came from Cancer Research UK (CRUKE/12/031) and an educational grant from Sanofi (Ref. No. Cabaz_L_05879). The trial was initiated and conducted independently by the trial investigators. The funders had no role in trial design, data collection, data analysis, data interpretation or writing of the report. The corresponding author had full access to all the data in the trial and had final responsibility for the decision to submit for publication.

## 3 RESULTS

**FIGURE 1:**
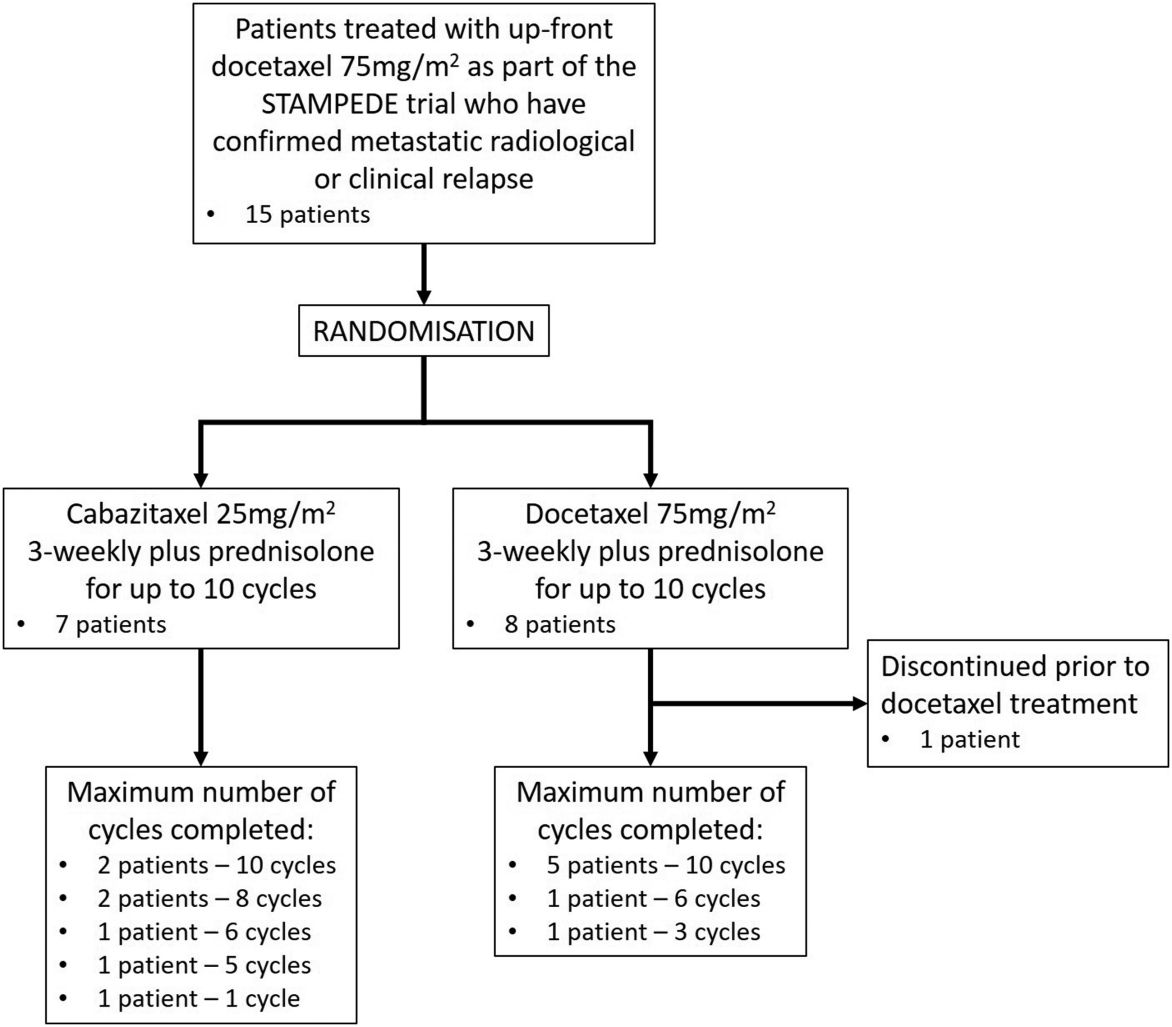
CANTATA trial profile.Consort diagram of the CANTATA trial

Between 7 March 2013 and 4 January 2016, 15 patients were randomised: seven to cabazitaxel and eight to docetaxel (Figure 1). The CANTATA trial failed to recruit to its pre‐specified target of 69 patients per arm and was halted due to slow accrual. No patients withdrew consent and none were lost to follow‐up. A descriptive analysis of the 15 evaluable patients recruited is described.

Patient characteristics and disease history at baseline are presented in Table 1. Baseline forms were returned by 11/15 patients (73.3%) with data demonstrating a median age for patients in the trial of 70 years (range 54–76). Patients in the cabazitaxel arm were younger than those in the docetaxel arm; 63 (interquartile rage [IQR]: 59.5–72.5) compared with 70 (IQR: 66.0–73.0), respectively. In addition, the majority of patients in the trial had received previous LHRH agonist therapy (10/15) and were taking concomitant medications (10/15) at baseline.

**TABLE 1:** Patient characteristics

	Cabazitaxel	Docetaxel	Total
	*N* = 7	*N* = 8	*N* = 15
Patient baseline characteristics, *n* (%)			
Age (years)			
Median	63	70	70
Interquartile range	59.5–72.5	66.0–73.0	64.0–73.0
Range	54–76	65–75	54–76
WHO performance status			
0	3 (42.9)	4 (50.0)	7 (46.7)
1	3 (42.9)	2 (25.0)	5 (33.3)
Missing	1 (14.3)	2 (25.0)	3 (20.0)
Concomitant medication			
No	0	1 (12.5)	1 (6.7)
Yes	6 (65.8)	4 (50.0)	10 (66.7)
Missing	1 (14.3)	3 (37.5)	4 (26.7)
Progression at study entry			
All	2 (28.6)	3 (37.5)	5 (33.3)
PSA	1 (14.3)	0	1 (6.7)
Radiological	1 (14.3)	0	1 (6.7)
PSA + radiological	2 (28.6)	3 (37.5)	5 (33.3)
Missing	1 (14.3)	2 (25.0)	3 (20.0)
Site of metastasis			
Bone	3 (42.9)	4 (50.0)	7 (46.7)
Distant node	1 (14.3)	0	1 (6.7)
Bone and lung	0	1 (12.5)	1 (6.7)
Bone and distant node	2 (28.6)	1 (12.5)	3 (20.0)
Missing	1 (14.3)	2 (25.0)	3 (20.0)
Prior therapy			
LHRH agonist	6 (85.8)	4 (50.0)	10 (66.7)
LHRH antagonist	0	1 (12.5)	1 (6.7)
LHRH agonist and antagonist	0	1 (12.5)	1 (6.7)
Missing	1 (14.3)	2 (25.0)	3 (20.0)
Pre‐existing adverse event			
No	4 (57.1)	4 (50.0)	8 (53.3)
Yes	1 (14.3)	0	1 (6.7)
Missing	2 (28.6)	4 (50.0)	6 (40.0)
Co‐morbidity			
No	2 (28.6)	3 (37.5)	5 (33.3)
Yes	4 (57.1)	2 (25.0)	6 (40.0)
Missing	1 (14.3)	3 (37.5)	4 (26.7)

Abbreviation: PSA, prostate‐specific antigen.

Seven (46.7%) patients received the full 10 cycles of treatment: two (28.6%) in the cabazitaxel arm and five (71.4%) in the docetaxel arm (Table 2). Eight (53.3%) patients received 10 changes to treatment dose with no patients receiving more than two reductions (as per protocol). Of these dose changes, seven (70%) were dose reductions, four (57.1%) patients receiving cabazitaxel and three (42.9%) receiving docetaxel. The most common reasons for patients receiving changes to their treatment were ‘other toxicity to protocol therapy’ (4/10) and myelosuppression (2/10). Two (14.3%) patients, both in the docetaxel arm, received a total of three dose escalations. No reasons were given for these.

**TABLE 2:** Treatments delivered

	Cycle									
Treatment	C1	C2	C3	C4	C5	C6	C7	C8	C9	C10
Cabazitaxel^a^										
*N*	7	6	6	6	6	5	4	4	2	2
Median	50	46	46	46	46	40	44	44	49	49
IQR	49.0–53.5	41.0–49.5	41.0–49.5	41.0–49.5	41.0–49.5	37.0–48.0	39.3–48.5	39.3–48.5	48.5–49.5	48.5–49.5
Range	47–55	37–50	37–50	37–50	37–50	0–50	37–50	37–50	48–50	48–50
Docetaxel^s^										
*N*	7^b^	7	7	6	6	6	5	5	5	5
Median	150.0	150.0	150.0	150.0	150.0	147.5	150.0	150.0	150.0	150.0
IQR	145.0–150.0	140.0–150.0	135.0–150.0	142.5–150.0	127.5–150.0	141.3–150.0	145.0–150.0	145.0–150.0	145.0–150.0	145.0–150.0
Range	130–150	130–150	110–150	75–150	110–150	120–150	120–150	120–150	120–150	120–150
Total	14	13	13	12	12	11	9	9	7	7
Intensity^c^										
Cabazitaxel	100	89	89	89	89	70	88	88	100	100
Docetaxel	100	99	96	92	93	96	96	96	96	96
Overall	100	94	93	90	91	84	92	92	97	97
Interrupted										
Yes	0	0	0	0	0	1	1	0	1	0
Reduced										
Yes	1	3	1	0	1	1	0	0	0	0
Escalated										
Yes	0	0	0	0	2	1	0	0	0	0
Discontinued										
Yes	1	0	1	0	2	1	0	2	0	0

Abbreviation: IQR, interquartile range.

^a^ Drug dose administered in mg/m^2^.

^b^ One patient withdrew prior to starting treatment.

^c^ Dose intensity given as a percentage of intended dose (actual dose/intended dose × 100).

One hundred and twenty‐nine concomitant medications were administered to patients during the trial (per patient range 1–80). Ninety‐four (71.3%) of these non‐pre‐existing concomitant medications were administered to patients in the cabazitaxel arm compared with 35 (27.1%) to patients in the docetaxel arm. The most common concomitant medications were dexamethasone (26/129), chlorphenamine (12/129) and ciprofloxacin (10/129).

**FIGURE 2:**
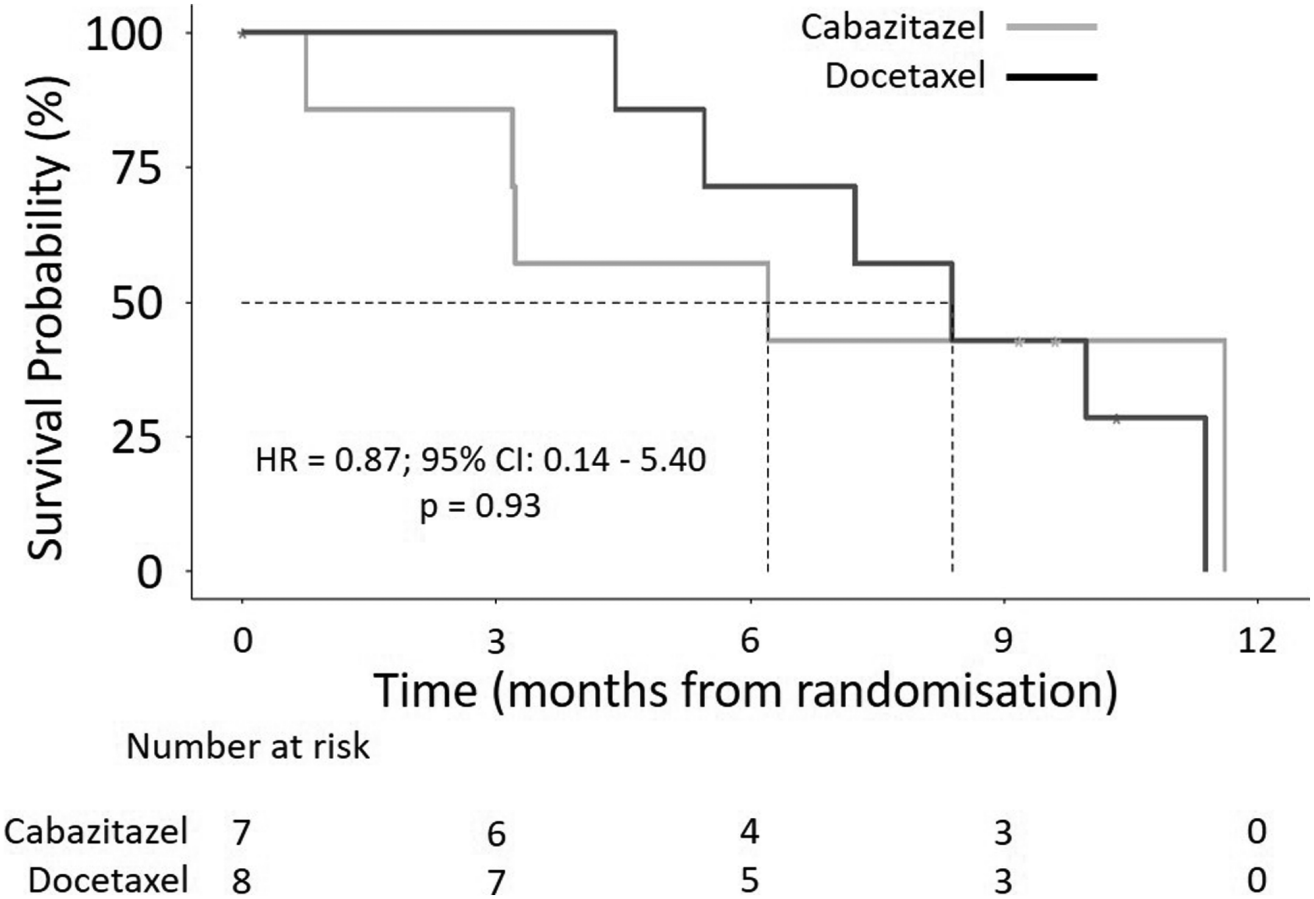
Clinical progression‐free survival. The primary outcome of clinical progression‐free survival defined as the earliest date of pain progression (the date a patient was seen in clinic and pain progression identified), date of occurrence of a cancer‐related skeletal‐related event or date of death from any cause. The dotted lines indicate median survival time. The adjusted hazard ratios (HR), 95% confidence intervals (CI) and *p* values were derived from Cox regression models.

The median follow‐up time in the cabazitaxel arm was 9.6 months from randomisation, and 10.3 months for the docetaxel arm. Median survival time for patients receiving cabazitaxel was 6.2 months compared with 8.4 for patients receiving docetaxel (95% CIs could not be reached due to the small number of patients) (Figure 2). The estimated clinical PFS percentages at 6 months were 57.1% (95% CI: 30.1–100) in the cabazitaxel arm, and 71.4% (95% CI: 44.7–100) in the docetaxel arm, with an overall percentage of 64.3 (95% CI: 43.5–100).

There were 402 adverse events reported during the CANTATA trial, of which, only 23 (5.7%) were Grade ≥3 (Appendix S2). The most common Grade ≥3 AE was diarrhoea (four occurrences). All patients in both arms experienced acute toxicity, with the tolerability of cabazitaxel 71.4% (5/7 experiencing Grade ≥3 haematological toxicities) compared with 42.9% (3/7) in the docetaxel arm. When the timing of occurrence of Grade ≥3 AEs was assessed, the majority of events in patients receiving cabazitaxel occurred within the first 2 cycles (10/14; 71.4%), compared with Cycles 3 and 4 for patients receiving docetaxel (5/9; 55.5%) (Table 3). Two late toxicities were reported in the cabazitaxel arm; alanine aminotransferase was increased in one patient 1 month after their last treatment was administered, and in another patient, dry mouth was reported 9.3 months after their last treatment was administered. Although no patients withdrew from the trial, eight patients discontinued treatment; five in the cabazitaxel arm and three in the docetaxel arm, including one patient (randomised to the docetaxel arm) who discontinued before commencing any treatment. The most common reason for discontinuation was toxicity (5/8).

**TABLE 3:** Timing of Grade ≥3 adverse events

	Cabazitaxel	Docetaxel	Total
	*N* = 14	*N* = 9	*N* = 23
Cycle number, *n* (%)			
1	7 (50.0)	0	7 (30.4)
2	3 (21.4)	0	3 (13.0)
3	0	2 (22.2)	2 (8.7)
4	0	3 (33.3)	3 (13.0)
5	3 (21.4)	1 (11.1)	4 (17.4)
6	1 (7.1)	2 (22.2)	3 (13.0)
7	0	0	0
8	0	0	0
9	0	0	0
10	0	1 (11.1)	1 (4.3)

Thirteen serious adverse events (SAEs) were reported, seven in the in the cabazitaxel arm and six in the docetaxel arm. Six were unrelated SAEs (two in the cabazitaxel arm, four in the docetaxel), and seven were serious adverse reactions (five in the cabazitaxel arm, two in the docetaxel). The SAEs occurred in nine patients, five of which had one SAE (three receiving cabazitaxel, two receiving docetaxel) and four patients experienced two SAEs (two receiving cabazitaxel, two receiving docetaxel).

**FIGURE 3:**
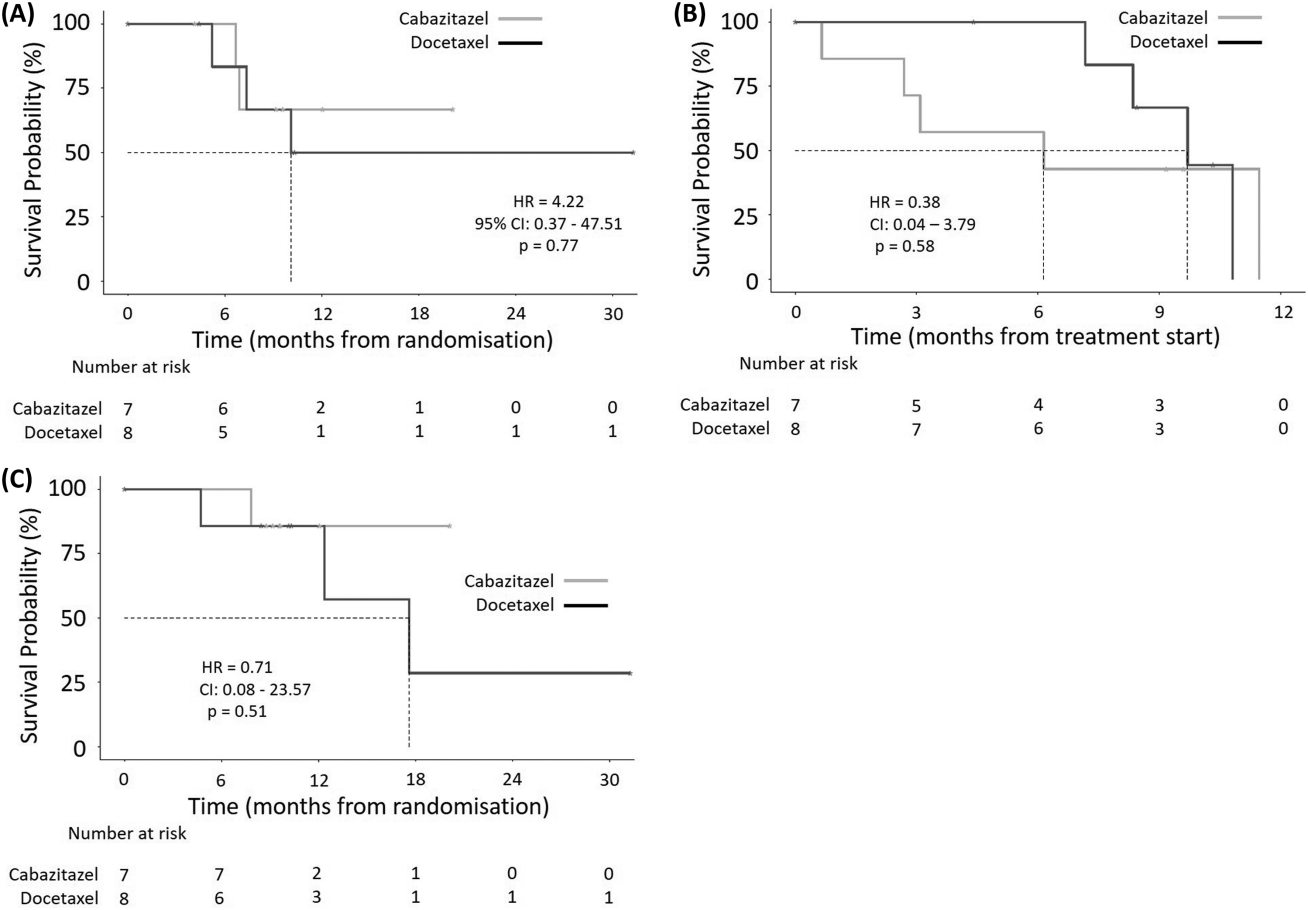
Secondary outcomes. Panel A shows the secondary outcome of pain progression‐free survival (PFS) defined as the time in whole days from the date of randomisation to the date of clinician‐determined pain progression. Patients not experiencing pain progression were censored at the date of death or their last known to be alive date. Panel B shows the secondary outcome of prostate‐specific antigen (PSA) PFS defined as a PSA decline of 50% confirmed by a second PSA value at least 3 weeks later measured from the start of initial treatment to the progression of PSA. Patients who did not experience PSA progression were censored at their date of death or at the date they were last known to be alive. Panel C shows the secondary outcome overall survival defined as the number of whole days from date of randomisation into the trial until death by any cause. Patients who did not die were censored at the date of last follow‐up. The dotted lines indicate median survival time. The adjusted hazard ratios, 95% confidence intervals and *p* values were derived from Cox regression models. CI, confidence interval; HR, hazard ratio.

No patients experienced any SREs for the duration of this trial. In addition, pain PFS could not be calculated in the cabazitaxel arm due to the small number of events. In the docetaxel arm, pain PFS was 10.1 months, with the estimated pain PFS percentage at 6 months 83.3% (95% CI: 58.3–100) (Figure 3A). Median PSA PFS time for the cabazitaxel and docetaxel arms were 6.2 and 9.7 months, respectively. The estimated PSA PFS percentages at 6 months from the date of randomisation were 57.1% (95% CI: 30.1–100) in the cabazitaxel arm, and 100.0% (95% CI: 100.0–100) in the docetaxel arm, with an overall percentage of 78.6 (95% CI: 59.8–100). (Figure 3B).

Median survival time could not be calculated in the cabazitaxel arm due to the small number of events. In the docetaxel arm, it was 17.6 months. The estimated overall survival probabilities at 6 months for the docetaxel arm was 85.7% (95% CI: 63.3–100.0) (Figure 3C).

A total of 13 (86.7%) patients completed at least one QoL form (seven receiving cabazitaxel and six receiving docetaxel), a median of 3 forms were completed during the trial (IQR: 2–3; range: 1–9). Baseline, end of treatment, and 3‐month post‐treatment forms had a completion rate of 78.6% across both arms. However, the 6‐month post‐treatment form was only completed by one patient in each arm, the 9‐month post‐treatment form by two patients receiving cabazitaxel and one patient receiving docetaxel, and forms for 12‐, 15‐, 18‐ and 21‐month post‐treatment completed by only one patient in the docetaxel arm. No overt differences in overall mean scores of the EORTC QLQ‐C30 and QLQ‐PR25 scores were observed between those patients receiving cabazitaxel compared with those receiving docetaxel (Appendix S3, Figures A and B).

## 4 DISCUSSION

The CANTATA trial was halted due to slow accrual. Therefore, we are unable to determine whether cabazitaxel can benefit clinical PFS compared with docetaxel re‐challenge as second‐line chemotherapy treatment in patients with mCRPC even though the question remains a relevant one given the positive proof of the role of upfront docetaxel in metastatic hormone‐sensitive prostate cancer (HSPC) demonstrated in the STAMPEDE^12^ and CHAARTED^13^ trials.

Despite the small number of patients randomised, we can still draw some conclusions from the data collected. Firstly, it is feasible and safe to deliver chemotherapy to men relapsing with CRPC after upfront chemotherapy for HSPC. The toxicity observed in the 15 patients treated was in line with that previously reported with these agents.^9^, ^11^ The overall median number of cycles received (8 and 10 for cabazitaxel and docetaxel, respectively) is also in line with the TAX327 trial of first‐line chemotherapy for CRPC.^9^ The median time to progression for each arm of 6–8 months is in line with TAX327 suggesting that these drugs are behaving like first‐line CRPC agents rather than second‐line treatments, where treatment effects are generally less. In comparison, in the TROPIC trial, the median PFS was only 2.8 months on cabazitaxel.^11^ In addition, although time to PSA progression is a different measure to the clinical PFS endpoint used in the CANTATA trial, the PROSELICA trial (comparing 20 with 25 mg/m^2^ cabazitaxel) time to PSA progression was 5.7–6.8 months again illustrating that the longer PFS observed is more in line with first‐line than second‐line chemotherapy.

The relative efficacies of docetaxel and cabazitaxel have been compared in first‐line chemotherapy in CRPC in the FIRSTANA trial in patients with no prior chemotherapy in HSPC.^21^ There were with no significant differences observed. Interestingly, the PFS durations of 4–5 months observed in this trial were less than seen in CANTATA although methods of measurement of PFS do vary between the two trials.

There are also no data from this trial to determine the impact of cabazitaxel, given post‐docetaxel, on third relapse. However, given that the two drugs are behaving in a similar fashion to first‐line chemotherapy in chemo‐naïve CRPC patients, it seems likely that further benefit from subsequent cabazitaxel would be observed as in the TROPIC trial.^11^ Although supported by limited data, it seems likely that the better chemotherapy strategy will be to use docetaxel on first relapse requiring chemotherapy, thereby keeping open the option of subsequent cabazitaxel.

No definitive conclusions on the relative efficacy of cabazitaxel and docetaxel in first‐line chemotherapy in patients relapsing after chemo‐hormonal therapy for HSPC can be drawn. The limited data obtained in CANTATA do, however, suggest that taxane chemotherapy in this setting performs similarly to first‐line chemotherapy in chemo‐naïve patients with CRPC. It thus seems reasonable to offer the standard CRPC sequence of docetaxel then cabazitaxel, thereby maximising therapeutic options for these patients.

## CONFLICT OF INTEREST

NDJ received grants from Cancer Research UK and Sanofi pertaining to this research. All other authors declare no competing interests.

## AUTHOR CONTRIBUTIONS

**Nicholas D. James** (Chief Investigator and first author): conception and design of the study, interpretation of data, drafting and review of paper. **Ayesha Ali**: trial statistician, analysis of data, interpretation of data, drafting and review of paper. **Ann Pope**: trial coordinator, interpretation of data, drafting and review of paper. **Amisha Desai**, **Daniel Ford**, **Robert Stevenson** and **Anjali Zarkar**: patient recruitment and treatment, drafting and review of paper. **Sarah Pirrie**: senior trial statistician, analysis of data, interpretation of data, drafting and review of paper.

## Supporting information


**Appendix S1.** Supporting Information

**Appendix S2.** Supporting Information

**Appendix S3.** Supporting Information

## Data Availability

Participant data and the associated supporting documentation will be available within 6 months after the publication of this manuscript. Details of our data request process are available on the CRCTU website. Only scientifically sound proposals from appropriately qualified research groups will be considered for data sharing. The decision to release data will be made by the CRCTU Director's Committee, who will consider the scientific validity of the request, the qualifications and resources of the research group, the views of the Chief Investigator and the trial steering committee, consent arrangements, the practicality of anonymising the requested data and contractual obligations. A data sharing agreement will cover the terms and conditions of the release of trial data and will include publication requirements, authorship and acknowledgements and obligations for the responsible use of data. An anonymised encrypted dataset will be transferred directly using a secure method and in accordance with the University of Birmingham's IT guidance on encryption of data sets.
