# Supplementary material for: Cabazitaxel versus docetaxel for treatment of metastatic castrate refractory prostate cancer
Source: BJUI Compass. 2022 Jun 18;3(6):484–93. doi: 10.1002/bco2.177 (PMC9579888; doi:10.1002/bco2.177)
Supplement: Supplementary file 1 — Appendix S1. Supporting Information [file BCO2-3-484-s002.docx]

# Supplementary Appendix 1: CANTATA Investigators

The CANTATA trial investigators include the following;

Prof R Jones, Beatson West of Scotland Cancer Centre

Dr A Hamid, Broomfield Hospital

Dr U Hofmann, Calderdale & Huddersfield Hospitals

Dr M Butt, Castle Hill Hospital

Dr S Hussain, Clatterbridge Cancer Centre

Dr S Beesley, Kent Cancer Centre

Dr S Sundar, Nottingham City Hospital

Prof N James, Queen Elizabeth Hospital, Birmingham

Dr P Chakraborti, Royal Derby Hospital

Dr D Sheehan, Royal Devon and Exeter Hospital

Dr S Dixit, Scunthorpe General Hospital

Prof J Wagstaff, Singleton Hospital

Dr N Srihari, The Shrewsbury and Telford Hospital NHS Trust

Dr A Lydon, Torbay Hospital

Dr J Tanguay, Velindre Cancer Centre
