# Supplementary material for: Cabazitaxel versus docetaxel for treatment of metastatic castrate refractory prostate cancer
Source: BJUI Compass. 2022 Jun 18;3(6):484–93. doi: 10.1002/bco2.177 (PMC9579888; doi:10.1002/bco2.177)
Supplement: Supplementary file 2 — Appendix S2. Supporting Information [file BCO2-3-484-s001.docx]

# Supplementary Appendix 2: Adverse Events

|  | Cabazitaxel | | Docetaxel | | Overall |  |
| --- | --- | --- | --- | --- | --- | --- |
| CTCAE category, v4.0 | *n* | % | *n* | % | *n* | % |
| Grade 4 symptoms |  |  |  |  |  |  |
| Acute kidney injury | 1 | 20.0 | 0 | 0.0 | 1 | 16.7 |
| Diarrhea | 2 | 40.0 | 0 | 0.0 | 2 | 33.3 |
| Fever | 1 | 20.0 | 0 | 0.0 | 1 | 16.7 |
| Hyperglycemia | 0 | 0.0 | 1 | 100 | 1 | 16.7 |
| Neutrophil count decreased | 1 | 20.0 | 0 | 0.0 | 1 | 16.7 |
| Total Grade 4 symptoms | 5 | 100 | 1 | 100 | 6 | 100 |
|  |  |  |  |  |  |  |
| Grade 3 symptoms |  |  |  |  |  |  |
| Anorexia | 1 | 11.1 | 0 | 0.0 | 1 | 5.9 |
| Atrial fibrillation | 1 | 11.1 | 0 | 0.0 | 1 | 5.9 |
| Back pain | 0 | 0.0 | 1 | 12.5 | 1 | 5.9 |
| Diarrhea | 2 | 22.2 | 0 | 0.0 | 2 | 11.8 |
| Fatigue | 1 | 11.1 | 1 | 12.5 | 2 | 11.8 |
| Febrile neutropenia | 1 | 11.1 | 1 | 12.5 | 2 | 11.8 |
| Hypotension | 0 | 0.0 | 1 | 12.5 | 1 | 5.9 |
| Lethargy | 0 | 0.0 | 1 | 12.5 | 1 | 5.9 |
| Lung infection | 0 | 0.0 | 1 | 12.5 | 1 | 5.9 |
| Neutrophil count decreased | 0 | 0.0 | 1 | 12.5 | 1 | 5.9 |
| Vomiting | 1 | 11.1 | 0 | 0.0 | 1 | 5.9 |
| Unknown | 2 | 22.2 | 1 | 12.5 | 3 | 17.6 |
| Total Grade 3 symptoms | 9 | 100 | 8 | 100 | 17 | 100 |
|  |  |  |  |  |  |  |
| Grade 2 symptoms |  |  |  |  |  |  |
| Alopecia | 0 | 0.0 | 7 | 30.4 | 7 | 11.5 |
| Anemia | 1 | 2.6 | 2 | 8.7 | 3 | 4.9 |
| Anorexia | 1 | 2.6 | 0 | 0.0 | 1 | 1.6 |
| Back pain | 0 | 0.0 | 1 | 4.3 | 1 | 1.6 |
| Bone pain | 1 | 2.6 | 0 | 0.0 | 1 | 1.6 |
| Chest pain - cardiac | 1 | 2.6 | 0 | 0.0 | 1 | 1.6 |
| Cough | 1 | 2.6 | 0 | 0.0 | 1 | 1.6 |
| Creatinine increased | 1 | 2.6 | 0 | 0.0 | 1 | 1.6 |
| Diarrhea | 1 | 2.6 | 0 | 0.0 | 1 | 1.6 |
| Dysgeusia | 1 | 2.6 | 0 | 0.0 | 1 | 1.6 |
| Fatigue | 13 | 34.2 | 4 | 17.4 | 17 | 27.9 |
| Febrile neutropenia | 1 | 2.6 | 1 | 4.3 | 2 | 3.3 |
| Fever | 2 | 5.3 | 1 | 4.3 | 3 | 4.9 |
| Gastrointestinal disorders | 1 | 2.6 | 0 | 0.0 | 1 | 1.6 |
| Gum infection | 0 | 0.0 | 1 | 4.3 | 1 | 1.6 |
| Hypotension | 0 | 0.0 | 1 | 4.3 | 1 | 1.6 |
| Lethargy | 1 | 2.6 | 1 | 4.3 | 2 | 3.3 |
| Lung infection | 1 | 2.6 | 0 | 0.0 | 1 | 1.6 |
| Malaise | 1 | 2.6 | 0 | 0.0 | 1 | 1.6 |
| Myalgia | 1 | 2.6 | 0 | 0.0 | 1 | 1.6 |
| Nausea | 1 | 2.6 | 1 | 4.3 | 2 | 3.3 |
| Neoplasms benign, malignant and unspecified | 1 | 2.6 | 0 | 0.0 | 1 | 1.6 |
| Neutrophil count decreased | 1 | 2.6 | 0 | 0.0 | 1 | 1.6 |
| Pain | 1 | 2.6 | 1 | 4.3 | 2 | 3.3 |
| Phlebitis | 1 | 2.6 | 0 | 0.0 | 1 | 1.6 |
| Skin infection | 0 | 0.0 | 1 | 4.3 | 1 | 1.6 |
| Sore throat | 1 | 2.6 | 0 | 0.0 | 1 | 1.6 |
| Urinary frequency | 1 | 2.6 | 0 | 0.0 | 1 | 1.6 |
| Ventricular arrhythmia | 1 | 2.6 | 0 | 0.0 | 1 | 1.6 |
| Vomiting | 1 | 2.6 | 1 | 4.3 | 2 | 3.3 |
| Total Grade 2 symptoms | 38 | 100 | 23 | 100 | 61 | 100 |
|  |  |  |  |  |  |  |
| Grade 1 symptoms |  |  |  |  |  |  |
| Acute kidney injury | 1 | 0.5 | 0 | 0.0 | 1 | 0.3 |
| Alanine aminotransferase increased | 4 | 2.2 | 0 | 0.0 | 4 | 1.3 |
| Alkaline phosphatase increased | 4 | 2.2 | 0 | 0.0 | 4 | 1.3 |
| Alopecia | 0 | 0.0 | 13 | 9.7 | 13 | 4.1 |
| Anemia | 21 | 11.4 | 6 | 4.5 | 27 | 8.5 |
| Anorexia | 3 | 1.6 | 4 | 3.0 | 7 | 2.2 |
| Arthralgia | 0 | 0.0 | 7 | 5.2 | 7 | 2.2 |
| Atrial fibrillation | 1 | 0.5 | 0 | 0.0 | 1 | 0.3 |
| Back pain | 2 | 1.1 | 7 | 5.2 | 9 | 2.8 |
| Blurred vision | 2 | 1.1 | 0 | 0.0 | 2 | 0.6 |
| Bone pain | 2 | 1.1 | 0 | 0.0 | 2 | 0.6 |
| Bruising | 6 | 3.3 | 0 | 0.0 | 6 | 1.9 |
| Chest pain - cardiac | 1 | 0.5 | 0 | 0.0 | 1 | 0.3 |
| Constipation | 1 | 0.5 | 5 | 3.7 | 6 | 1.9 |
| Cough | 1 | 0.5 | 0 | 0.0 | 1 | 0.3 |
| Creatinine increased | 1 | 0.5 | 0 | 0.0 | 1 | 0.3 |
| Depression | 1 | 0.5 | 0 | 0.0 | 1 | 0.3 |
| Diarrhea | 10 | 5.4 | 16 | 11.9 | 26 | 8.2 |
| Dry mouth | 2 | 1.1 | 0 | 0.0 | 2 | 0.6 |
| Dry skin | 0 | 0.0 | 1 | 0.7 | 1 | 0.3 |
| Dysgeusia | 9 | 4.9 | 2 | 1.5 | 11 | 3.5 |
| Dyspnea | 8 | 4.3 | 2 | 1.5 | 10 | 3.1 |
| Ear pain | 1 | 0.5 | 1 | 0.7 | 2 | 0.6 |
| Epistaxis | 5 | 2.7 | 0 | 0.0 | 5 | 1.6 |
| Fatigue | 26 | 14.1 | 22 | 16.4 | 48 | 15.1 |
| Febrile neutropenia | 2 | 1.1 | 2 | 1.5 | 4 | 1.3 |
| Fever | 3 | 1.6 | 1 | 0.7 | 4 | 1.3 |
| Gastrointestinal disorders | 3 | 1.6 | 3 | 2.2 | 6 | 1.9 |
| General disorders and admin site conditions | 1 | 0.5 | 1 | 0.7 | 2 | 0.6 |
| Gum infection | 0 | 0.0 | 1 | 0.7 | 1 | 0.3 |
| Headache | 1 | 0.5 | 0 | 0.0 | 1 | 0.3 |
| Hot flashes | 1 | 0.5 | 0 | 0.0 | 1 | 0.3 |
| Hyperglycemia | 0 | 0.0 | 1 | 0.7 | 1 | 0.3 |
| Hyperkalemia | 1 | 0.5 | 0 | 0.0 | 1 | 0.3 |
| Hypoalbuminemia | 10 | 5.4 | 0 | 0.0 | 10 | 3.1 |
| Hypokalemia | 3 | 1.6 | 0 | 0.0 | 3 | 0.9 |
| Hypotension | 0 | 0.0 | 2 | 1.5 | 2 | 0.6 |
| Investigations | 2 | 1.1 | 0 | 0.0 | 2 | 0.6 |
| Lethargy | 5 | 2.7 | 5 | 3.7 | 10 | 3.1 |
| Lung infection | 1 | 0.5 | 1 | 0.7 | 2 | 0.6 |
| Malaise | 1 | 0.5 | 0 | 0.0 | 1 | 0.3 |
| Mucositis oral | 2 | 1.1 | 0 | 0.0 | 2 | 0.6 |
| Muscle weakness lower limb | 0 | 0.0 | 1 | 0.7 | 1 | 0.3 |
| Muscle weakness upper limb | 0 | 0.0 | 1 | 0.7 | 1 | 0.3 |
| Myalgia | 3 | 1.6 | 0 | 0.0 | 3 | 0.9 |
| Nail ridging | 0 | 0.0 | 4 | 3.0 | 4 | 1.3 |
| Nausea | 3 | 1.6 | 5 | 3.7 | 8 | 2.5 |
| Neoplasms benign, malignant and unspecified | 1 | 0.5 | 0 | 0.0 | 1 | 0.3 |
| Nervous system disorders | 0 | 0.0 | 2 | 1.5 | 2 | 0.6 |
| Neutrophil count decreased | 2 | 1.1 | 1 | 0.7 | 3 | 0.9 |
| Pain | 1 | 0.5 | 1 | 0.7 | 2 | 0.6 |
| Peripheral sensory neuropathy | 4 | 2.2 | 3 | 2.2 | 7 | 2.2 |
| Phlebitis | 1 | 0.5 | 0 | 0.0 | 1 | 0.3 |
| Platelet count decreased | 2 | 1.1 | 0 | 0.0 | 2 | 0.6 |
| Respiratory, thoracic and mediastinal disorders | 1 | 0.5 | 0 | 0.0 | 1 | 0.3 |
| Sinusitis | 1 | 0.5 | 0 | 0.0 | 1 | 0.3 |
| Skin and subcutaneous tissue disorders | 1 | 0.5 | 1 | 0.7 | 2 | 0.6 |
| Skin infection | 0 | 0.0 | 2 | 1.5 | 2 | 0.6 |
| Sore throat | 2 | 1.1 | 0 | 0.0 | 2 | 0.6 |
| Tumor pain | 0 | 0.0 | 1 | 0.7 | 1 | 0.3 |
| Urinary frequency | 1 | 0.5 | 0 | 0.0 | 1 | 0.3 |
| Urinary tract infection | 0 | 0.0 | 1 | 0.7 | 1 | 0.3 |
| Ventricular arrhythmia | 1 | 0.5 | 0 | 0.0 | 1 | 0.3 |
| Vomiting | 4 | 2.2 | 2 | 1.5 | 6 | 1.9 |
| Watering eyes | 1 | 0.5 | 2 | 1.5 | 3 | 0.9 |
| Weight loss | 0 | 0.0 | 1 | 0.7 | 1 | 0.3 |
| Unknown | 7 | 3.8 | 3 | 2.2 | 10 | 3.1 |
| Total Grade 1 symptoms | 127 | 100 | 101 | 100 | 228 | 100 |
|  |  |  |  |  |  |  |
| TOTAL ALL GRADE SYMPTOMS | 236 |  | 166 |  | 402 |  |
