# Supplementary material for: Cabazitaxel versus docetaxel for treatment of metastatic castrate refractory prostate cancer
Source: BJUI Compass. 2022 Jun 18;3(6):484–93. doi: 10.1002/bco2.177 (PMC9579888; doi:10.1002/bco2.177)

# Supplementary Appendix 3: Quality of Life

The overall mean scores from the EORTC QLQ-C30 and EORTC QLQ-PR25 are shown in panels A and B, respectively. Due to the small sample size, 95% confidence intervals have been omitted for clarity.


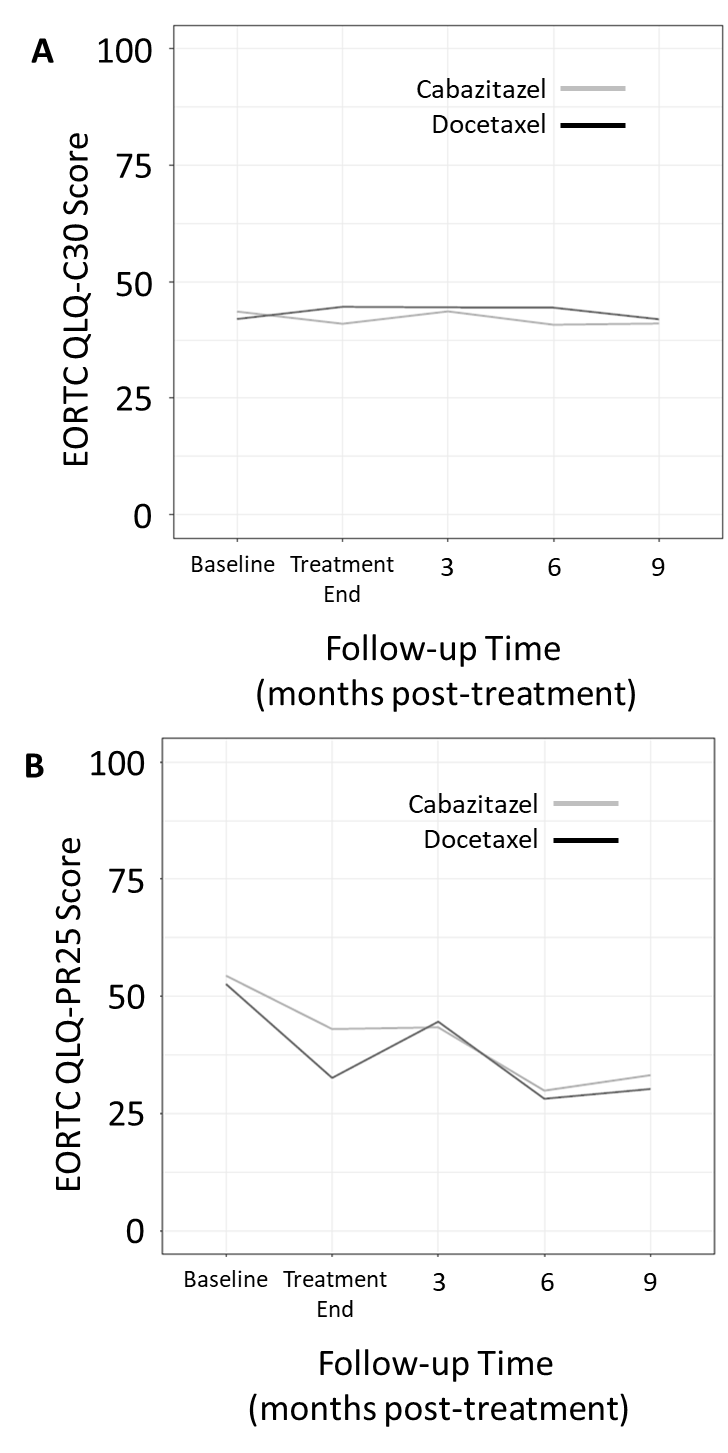

Supplement: Supplementary file 3 — Appendix S3. Supporting Information [file BCO2-3-484-s003.docx]
